# Pulmonary Proteome and Protein Networks in Response to the Herbicide Paraquat in Rats

**DOI:** 10.4172/jpb.1000354

**Published:** 2015-05

**Authors:** Il Kyu Cho, Mihye Jeong, Are-Sun You, Kyung Hun Park, Qing X. Li

**Affiliations:** 1 Department of Molecular Biosciences and Bioengineering, University of Hawaii at Manoa, Honolulu, HI 96822, USA; 2 Department of Agro-Food Safety, National Academy of Agricultural Science, Rural Development Administration, Chonbuk 565-851, Republic of Korea

**Keywords:** Paraquat, Pesticide exposure, Proteome, Pulmonary proteins, Ingenuity pathway analysis, p53 signaling

## Abstract

Paraquat (PQ) has been one of the most widely used herbicides in the world. PQ, when ingested, is toxic to humans and may cause acute respiratory distress syndrome. To investigate molecular perturbation in lung tissues caused by PQ, Sprague Dawley male rats were fed with PQ at a dose of 25 mg/kg body weight for 20 times in four weeks. The effects of PQ on cellular processes and biological pathways were investigated by analyzing proteome in the lung tissues in comparison with the control. Among the detected proteins, 321 and 254 proteins were over-represented and under-represented, respectively, in the PQ-exposed rat lung tissues in comparison with the no PQ control. All over- and under-represented proteins were subjected to Ingenuity Pathway Analysis to create 25 biological networks and 38 pathways of interacting protein clusters. Over-represented proteins were involved in the C-jun-amino-terminal kinase pathway, caveolae-mediated endocytosis signaling, cardiovascular-cancer-respiratory pathway, regulation of clathrin-mediated endocytosis, non-small cell lung cancer signaling, pulmonary hypertension, glutamate receptor, immune response and angiogenesis. Under-represented proteins occurred in the p53 signaling pathway, mitogen-activated protein kinase signaling pathway, cartilage development and angiogenesis inhibition in the PQ-treated lungs. The results suggest that PQ may generate reactive oxygen species, impair the MAPK/p53 signaling pathway, activate angiogenesis and depress apoptosis in the lungs.

## Introduction

Paraquat (1,1-dimethyl-4,4′-bispyridinium dichloride) has been one of the most widely used herbicides in the world since its introduction to the market in 1962 [1]. It is very toxic to mammals including humans and ingestion may cause acute respiratory distress syndrome. Paraquat accumulates in the human lungs at a concentration 10 times greater than that in the other tissues [2]. It has been reported that PQ induces pulmonary fibrosis in humans, monkeys, dogs, and rats [3–9]. The major toxicity is known to cause damages in the tissues of lungs, livers and kidneys. It particularly makes lungs harden and causes severe lung injuries, such as interstitial edema, leukocyte infiltration, alveolar hemorrhage edema, fibroblast proliferation and collagen deposition when it is accumulated in lungs [10–12]. Paraquat serves as a catalyst to transfer electrons from NADPH to oxygen molecules to produce superoxide that breaks up cell membranes and tissues [13]. Human toxicity from PQ exposure takes place in two phases. The initial phase ends in death of pulmonary edema, cell infiltration and alveolar hemorrhage within a few days. People surviving the destructive phase may progress to the second proliferative phase, which lasts for several weeks [14]. Infiltration and proliferation of fibroblasts may cause fibrosis that eradicates alveolar tissue. No successful treatment has passed clinical trials although many treatment protocols for PQ poisoning have been studied [15–18].

PQ has been evaluated as an environmental factor that could be involved in the etiology of Parkinson’s disease, which is caused by a disorder affecting the dopamine neurons in the brain [19–21]. Involvements of dopamine and glutamate in the neural toxicity of PQ related to Parkinson’s disease have been proposed [6,22]. In 2011, a study funded by the U.S. National Institutes of Health showed a link between PQ exposure and Parkinson’s disease in farm workers [23].

Early diagnostic biomarkers are important for treatment of acute PQ poisoning. In order to investigate the characterization of diagnostic biomarkers in PQ poisoning, Kim et al. [24] employed a 2D-PAGE MALDI-TOF/TOF approach and identified that apolipoprotein E (ApoE), a precursor of haptoglobin (Hp) and fibrinogen γ-chain (FGG) were potential biomarkers of PQ exposure in rat sera. In an effort to develop a remedy for PQ poisoning, Kim et al. [25] conducted proteomic analysis of PQ-poisoned male CD(SD)IGS rat lungs to identify any protective effects of acai berry on the PQ poisoning.

The objectives of this study were to measure protein profiles in response to PQ and understand the responded protein networks in rats exposed to PQ. To our knowledge, this is the first comprehensive profile of soluble proteins in the lungs of rats treated with low levels of PQ that would not cause differences in the body weight gain, hematological parameters and clinic chemistry.

## Materials and Methods

### Rats

Eight week-old Sprague Dawley Crj:CD 1GS male rats were obtained from Charles River Japan, Inc. (Yokohama, Kanagawa 222-0033). The rats were maintained according to the protocol approved by the Korean Rural Development Administration, Korea. The rats were housed at a temperature of 23 ± 2°C, humidity of 55 ± 5% and luminance of 200~300 Lux in a constant 12 h light/12 h dark cycle. They were supplied with autoclaved water and rat chow (Samyang Co., Seoul, Korea). The rats were individually weighed at the beginning of the experiment and thereafter regularly observed for body weight, feed and water intakes for 28 days. The lung weight and hematology were observed after a postmortem examination. Body weight, hematological test and clinic chemistry data were assessed by one-way ANOVA of the GraphPad Prism Version 5.00 (GraphPad Software, San Diego, USA).

### Paraquat administration

The no observable effect level (NOEL), lowest-observed-adverse-effect level (LOAEL) and LD_50_ of paraquat dichloride (MW, 276) to Sprague Dawley male and female rats are 7 mg/kg bw/day, 20 mg/kg bw/day and 150 mg/kg, respectively [26–28]. PQ (Sigma, St. Louis, MO) was dissolved in saline (100 mg/kg) and orally administered via trans-oral at dosages of 0 (saline control), 5, 10, 25 and 40 mg/kg bw to five groups of 10 male rats per group five times a week for four weeks based on a 13-week oral toxicity study [4,27]. Of the four treatment levels, the lungs of rats orally dosed with 25 mg/kg bw PQ, which is at approximately PQ LOAEL (20 mg/kg bw/day) and six times below LD_50_ (150 mg/kg), were used for profiling soluble proteins in this study.

### Lung tissue samples

After animals were sacrificed, no visible symptoms of toxicity were observed in any organs and tissues. The lung samples were collected from the control rats and the rats dosed with 25 mg/kg bw PQ for the proteomic studies, but not the other groups. The rats were anesthetized with sodium pentobarbital (50 mg/kg) via intraperitoneal (i.p.) injection prior to dissection. After quick removal of entire lungs, the lower half of the right lung was isolated and frozen in liquid nitrogen. The specimens were stored at −80°C until protein extraction. Lung samples from three rats of each group were randomly selected for protein extraction and analysis.

### Protein samples

The treatment with 25 mg/kg bw PQ did not cause difference in the bw gain, hematological parameters and clinic chemistry. The lung sample from each rat was ground in liquid N_2_ using a mortar and pestle. One hundred mg of the powdered lung tissues (wet weight) was extracted with 1.75 mL of a mixture of 1 mL lysis buffer and 0.75 mL protease inhibitor cocktail (Roche Diagnostics, Basel, Switzerland) under ultrasonication (Microson^™^ ultrasonic cell disruptor, Misonix, Farmingdale, NY) at speed level 1 for 15 min at ambient temperature. The lysis buffer contained 40 mM tris-HCl, pH 7.4, 5 mM dithiothreitol (DTT) and 1 mM phenylmethylsulfonyl fluoride. The homogenate was then centrifuged at 17,000 *g* for 30 min. The supernatant was filtered through a membrane Econofilter (0.2 mm×25 μm, Agilent, Palo Alto, CA) and then used for protein analysis. Protein concentrations were determined with Coomassie Plus^™^ protein assay kit (Pierce, Rockford, IL).

### One dimensional (1D) SDS-PAGE, protein digestion and peptide extraction

Lung sample solutions from the control (3.5 μL) and the 25-mg/kg bw treatment (3.9 μL) (both 25 μg protein equivalents) were separately mixed with SDS-PAGE sample buffer (3.5 μL and 3.9 μL, respectively) and heated at 100°C for 5 min. The denatured proteins were separated on 10–20% gradient SDS-PAGE mini gels (9×10 cm, PAGE Gold Precast Gel, Cambrex Bioscience, Rockland, ME) followed by Coomassie dye (G-250) staining. Protein molecular weight (MW) was estimated with Precision plus protein standards (10 μL) (Bio-Rad, Hercules, CA) on the gels (Supplemental data Figure 1). Each gel lane was cut into 20 even slices, destained with 50% (v/v) acetonitrile (ACN) in 25 mM NH_4_HCO_3_, and then completely dried in a speed-vacuum centrifuge (vacufuge^™^, Eppendorf, Hamburg, Germany) after dehydration with ACN. The dried gel slices were reduced in 50 μL of 10 mM DTT for 45 min at 56°C, alkylated in 50 μL of 55 mM iodoacetamide for 45 min at ambient temperature in the dark. The gel slice samples were dehydrated with ACN followed by drying in the speed-vacuum centrifuge. After addition of 20 μL of sequencing-grade modified porcine trypsin (20 ng/μL in 50 mM NH_4_HCO_3_), samples were incubated at 37°C overnight. Tryptic digestion was stopped by adding 5 μL of 2% trifluoroacetic acid (TFA). The digested peptides were extracted twice from each gel slice with 30 μL of water/ACN/TFA (93:5:2, v/v/v) by sonication for 10 min in an ice bath and then combined for protein analysis.

### Protein identification

Digested peptides of which the proteins were separated on 1D gel were analyzed on a Dionex UltiMate^™^ 3000 LC interfaced with a Bruker esquireHCT^ultra^ ion trap mass spectrometer (Bruker Daltonics, Billerica, ME) in nanoelectrospray mode with a PicoTip Emitter (360 μm O.D., 20 μm I.D., 10 μm tip I.D., New Objective, Woburn, MA) according to the procedure previously described [29,30]. Mascot-peptide mass fingerprinting (PMF) searches and sequence alignments were performed with the Swiss-Prot databases. UniProt classification was used to search cellular roles of identified proteins. Peptides were assumed to be monoisotopic, oxidized at methionine residues and carbamidomethylated at cysteine residues. Up to one missed trypsin cleavage was allowed, although matches that contained any missed cleavages were not noticed. Peptide mass and MS/MS tolerances were set at ± 1.0 and ± 0.8 Da, respectively. Probability-based molecular weight search (MOWSE) scores were estimated and were reported as: 10 × log_10_ (*p*), where *p* is the absolute probability. Scores in Mascot larger than the MOWSE score at *p*=0.05 were considered statistically significant, meaning that the probability of the match being a random event is lower than 0.05. The false-positive rate (FPR) was estimated [31] and was smaller than 2% [FPR=FP/(FP+TP), where FP is the number of FPR hits; TP is the number of true-positive hits]. Only proteins identified with at least two peptide hits in triplicate analyses, with each peptide containing two tryptic termini, were accepted. In addition, the MS/MS spectra of all positively identified peptides were manually confirmed twice. Proteins detected in the treatment samples were compared with those in the saline control samples. Detection of a protein in the treatment sample but not in the control is referred to as over-representation, whereas absence of a protein in the treatment sample but presence in the control is referred to as under-representation. Only proteins detected in all three rat replicates were considered.

### Pathway and network analysis

Accession numbers of detected proteins (UniProt/SwissProt Ids) were listed in MS Excel and then imported into the Ingenuity Pathway Analysis (IPA) (Ingenuity System, Redwood City, CA) to create canonical pathways and networks of interacting proteins. Protein IDs uploaded into IPA were converted to the corresponding rat Entrez Gene symbols for display in pathways and networks. The data were analyzed in the context of rat (IPA ID No.: 156502). The network scores [32–55] were based in the hyper geometric distribution and were calculated with the right-tailed Fisher’s Exact Test. The scores were not an indication of the quality or biological relevance of the network. They simply measured the approximate “fit” between each network and the network eligible molecules. The p values were associated with a function or a pathway in Global Functional Analysis (GFA) and Global Canonical Pathway (GCP).

## Results

### Body weight changes

All rats gradually gained body weights during the four week experiment period (Supplemental data Table 1). The body weights of the PQ-orally treated rats were not significantly different from those of control rats, although food and water consumption was slightly decreased in all PQ-treated rats in comparison with the control rats (data not shown). It is noteworthy that additional rats were also administered with PQ via i.p. or subcutaneous injections at the dose levels of 5, 10, 25 and 40 mg/kg bw, however, those rats gained significantly less weights than the PQ-orally treated rats and the saline control rats (data not shown).

### Hematological test and clinic chemistry of rats

No significant difference was found between all PQ-treated rat groups and the control group for the hematological parameters (Supplemental data Table 2) and for the clinic chemistry (Supplemental data Table 3).

### Differentially expressed proteins

**Figure 1:**
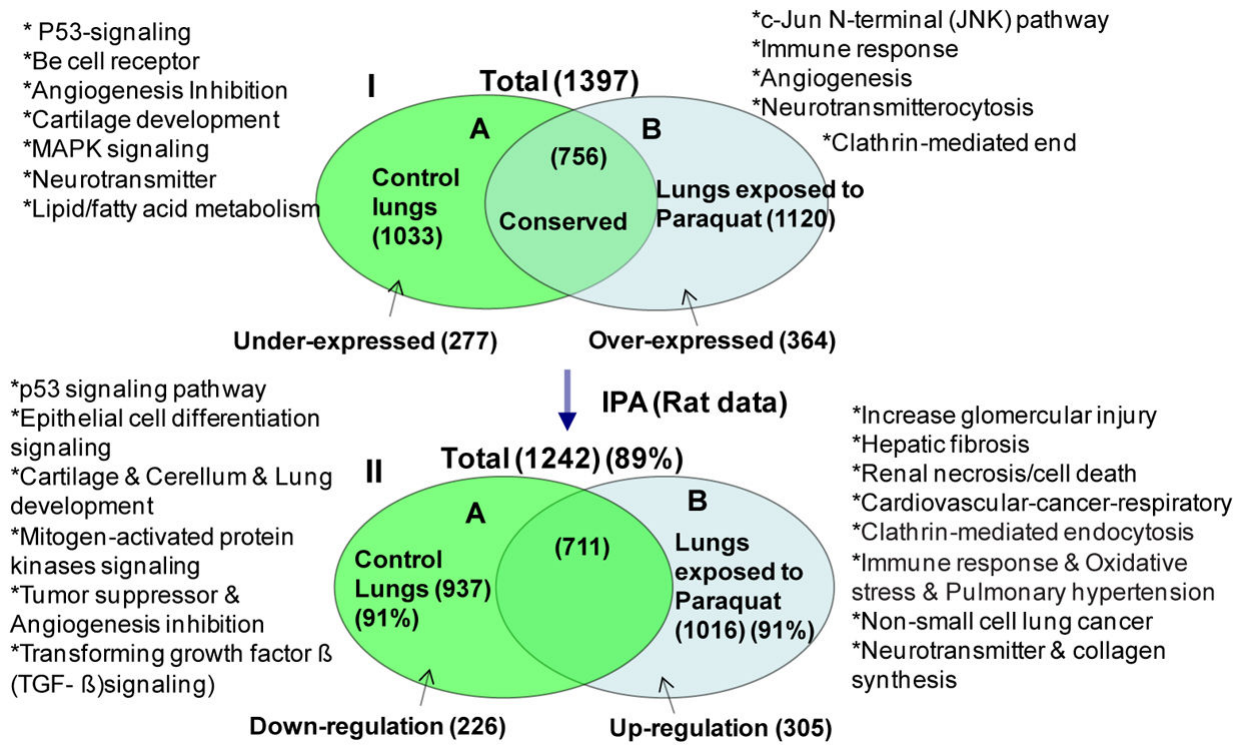
Venn diagrams showing 1033 and 1120 proteins detected (I), 937 and 1016 proteins mapped (II) in the lungs of the control and PQ-treated rats, respectively. The number of proteins detected was indicated in the parentheses.

A total of 1033 and 1120 proteins was detected in the lungs of the control rats and PQ-exposed rats, respectively, while 756 of these proteins were detected in both groups (Figure 1, I, Supplemental data Table 4 and Table 5). There were 254 and 321 proteins under- and over-represented, respectively, in the lungs of rats treated with PQ at 25 mg/kg bw as compared with the control rats. Among the 254 under-represented proteins, 65 proteins that had 8 or more matched peptides per protein, Mascot scores higher than 140 and known biological functions were considered as major under-represented proteins (Table 1). Among the 321 over-represented proteins, 57 proteins that had 12 or more matched peptides per protein, Mascot scores higher than 134 and known biological functions were considered as major over-represented proteins (Table 2).

#### Under-represented proteins

Table 1 shows that major under-represented proteins involved in p53 signaling and MAPK signaling pathways, apoptosis, angiogenesis inhibition and B cell receptor complex. The major under-represented proteins involved in p53 signaling pathway include TP53BP1, BCL11B, DPH1, HIC1 and CHTF8 in the lungs of rats treated with 25 mg/kg bw of PQ. However, these proteins were detected in the lung tissues of the control rats.

#### Over-represented proteins

The over-represented proteins include DUSP10, MAPK8IP2, CASR and MAP3K1 (Table 2). These proteins are related to the c-Jun N-terminal kinase (JNK) pathway, which is also known as stress-activated protein kinase (SAPK) [32–34]. It plays a role on responses to diverse environmental stresses [35–37]. Over-represented proteins associated with immune response and antioxidant defense included MARCO, NLRX1, FAIM3, C1orf38, Cd55/Daf2, BCL3, TXNRD3 and RNF125 in the lungs of rats treated with PQ. Macrophages are regulators of homeostasis and effecter cells in infection wounding and tumor growth in the immune system [38].

The findings indicated pulmonary stresses and activation of antioxidant defense system and immune system responses in rat lungs against adverse effects after PQ exposure [10,39,40]. Angiogenesis-related proteins such as roundabout homolog 4, ETS domain-containing protein Elk-3, collagen alpha-2 (IV) chain and nitric oxide synthase were over-represented in the lung tissues from rats treated with PQ. In addition, glutamate receptor (ionotropic, metabotropic 5, metabotropic 1) proteins were over-represented in the rat lungs.

### Protein networks

**Figure 2:**
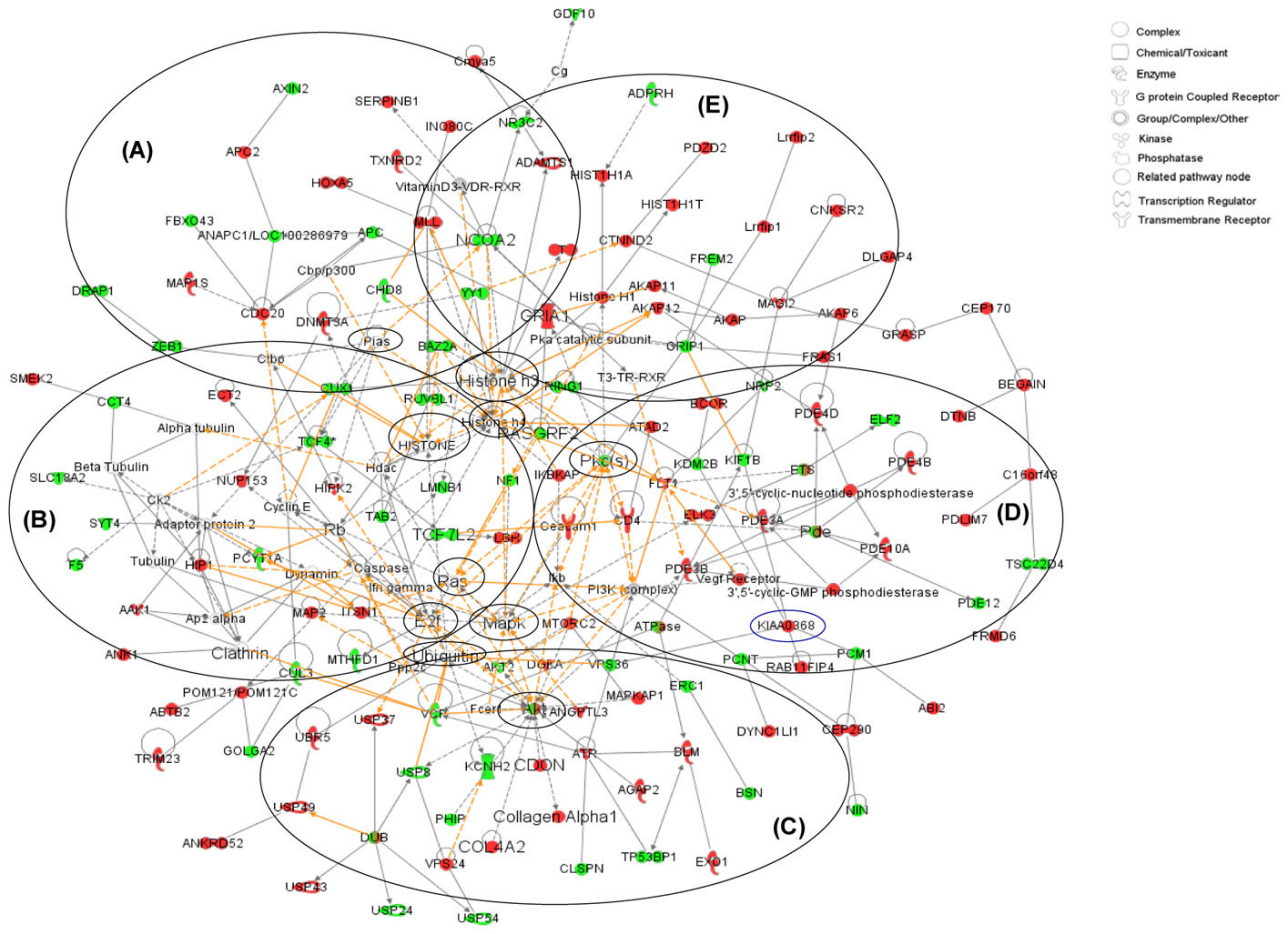
Five sub-networks were highlighted with thick circles. Eleven small circles identified proteins that also served as hubs for groups of proteins. Proteins in red were the over-represented, while proteins in green were under-represented. Solid grey lines and dotted grey lines indicated direct and indirect interactions, respectively. Arrow-headed lines and simple lines without arrows indicated ‘acts on’ and ‘binding only’, respectively. Solid orange lines and dotted orange lines indicated highlighted direct and indirect interactions, respectively. Lines connecting the proteins indicated known interrelationships from the IPA database.

Out of the 254, 321 and 756 of under-represented, over-represented and overlapped proteins, 226, 305 and 711 identifies (IDs), respectively, were mapped into 25 biological networks and 72 pathways of interacting protein clusters according to the identifiers’ HomoloGene to the ortholog information in the Ingenuity Knowledge Base (IKB) (Rat data) (Figure 1, II). The IKB contains published findings for both genes and proteins. However, for display purposes, only gene names were used, even when proteins IDs were uploaded. The biological network of differentially expressed proteins showed inter-relationships and relevant signaling pathways (Figure 2). One overall network was merged from the 5 highest scored (scores, 30–53) networks (A, cell-to-cell signaling and interaction; B, cell signaling; C, cell cycle: D, genetic disorder: E, cellular movement). In the network, interrelationships of proteins linked to central hubs (11 small circles in Figure 2) present cellular assembly and organization, cellular function and maintenance, developmental disorder. The network showed that a fibrosis is correlated with the expression of an alpha-1 collagen (Figure 2) in the PQ-treated rat lungs.

**Figure 3:**
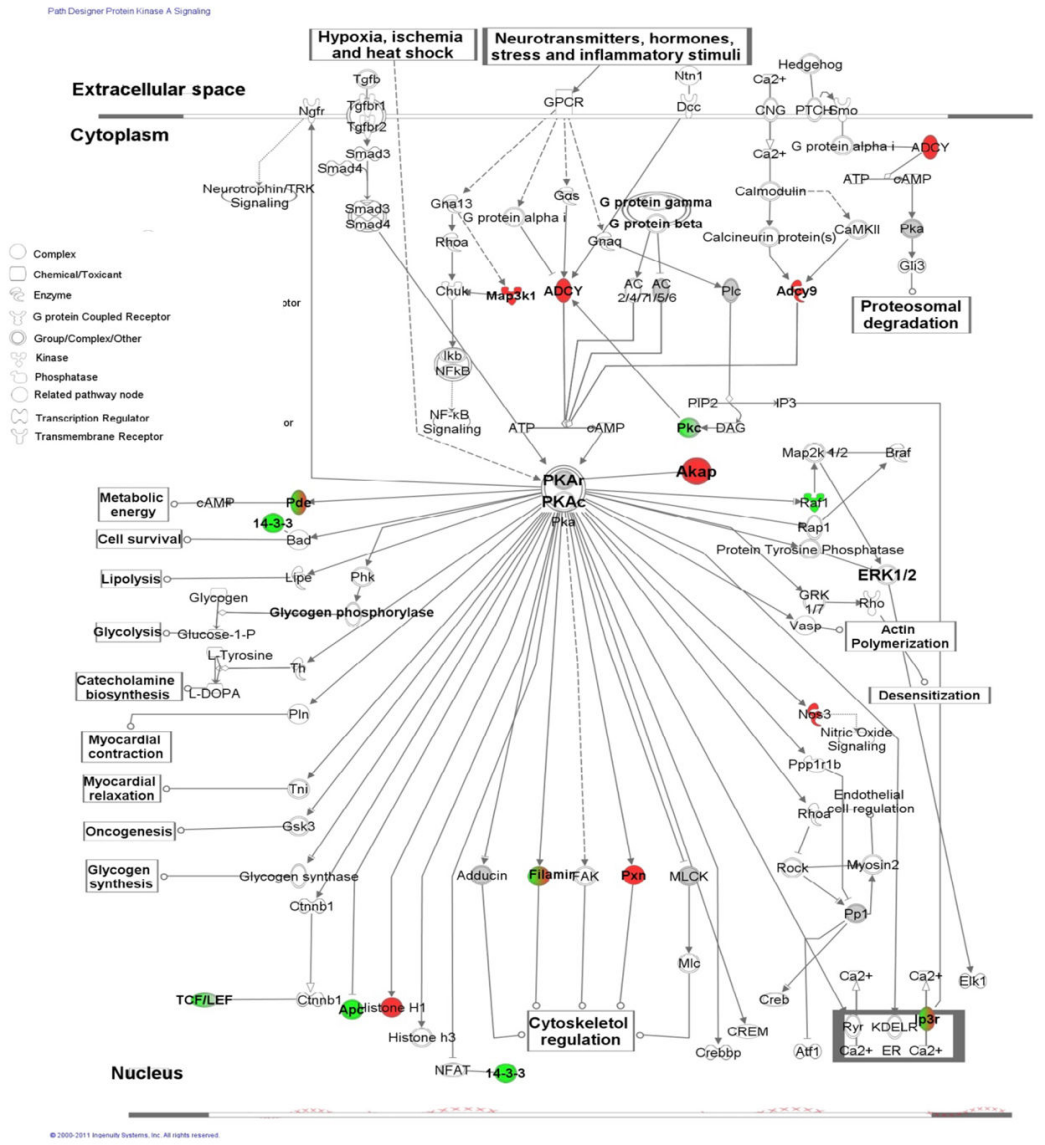
Up-regulation of protein kinase A signaling in the lungs of PQ-treated rats. Proteins in red were the over-represented, while proteins in green were the under-represented. Proteins in half green and half red were detected in both the PQ-free and PQ-treated rat lungs. Proteins in white and grey indicated that the proteins are related to the signaling, but were not detected. In addition, proteins in grey were hubs

Table 3 presents top five up- and down-regulated network functions, canonical pathways, toxicity (tox) lists and functions in rat lung tissues in response to PQ exposure. Twenty five networks and 42 pathways were up-regulated in the lungs of PQ-treated rats, whereas 25 networks and 54 pathways were down-regulated. The top up-regulated canonical pathways in which many over-represented proteins were detected were protein kinase A signaling (Figure 3), caveolae-mediated endocytosis signaling (Supplemental data Figure 2), EGF signaling, GNRH signaling and cardiac β-adrenergic signaling (Table 3). The top down-regulated canonical pathways in which under-represented proteins were detected were putative molecular mechanisms of cancer (Supplemental data Figure 3), acute myeloid leukemia signaling, erythropoietin signaling, mechanisms of viral exit from host cells and IL-3 signaling (Table 3). The top tox lists were increase of adenylate cyclase, hepatic fibrosis, renal necrosis/cell death, TR/RXR activation and PXR/RXR activation (Table 3). The top down-regulated tox lists included VDR/RXR activation, G2/M DNA damage checkpoint regulation, cardiac fibrosis, PPARα/RXRα activation and p53 signaling (Table 3). Top up-regulated functions in the lungs of PQ-treated rats were 1) cardiovascular-cancer-respiratory, which is related to respiratory-nitric oxide synthase (NOS3), 2) clathrin-mediated endocytosis, which is regulated by AP2-associated protein kinase (AAK1), 3) immune response/oxidative stress/pulmonary hypertension, 4) non-small cell lung cancer, which comprises about 80% of all lung cancers, 5) neurotransmitter/collagen synthesis (Table 3). Top down-regulated functions were 1) epithelial cell differentiation signaling, 2) cartilage/cerellum/lung development, 3) mitogen-activated protein kinases signaling, 4) transforming growth factor β (TGF-β) signaling, and 5) tumor suppressor. The results of the present study supported the previous studies of which PQ might induce pulmonary fibrosis, e.g., up-regulation of neurotransmitter, collagen synthesis and oxidative stress and down-regulation of epithelial cell differentiation, lung development and TGF-β signaling, cellular reactive oxygen species (ROS) in the PQ-treated rat lungs [4,6,8,9,11,41].

### Up-regulation of protein kinase A signaling in the lungs of PQ-treated rats

Protein Kinase A (PKA) signaling was found to be the highest dominant canonical pathway (Figure 3). PKA regulates processes as diverse as growth, development, memory and metabolism [40]. It exists as a tetramer complex of two catalytic subunits (PKA-C) and a regulatory (PKA-R) subunit dimmer. Twenty six proteins were associated with the PKA signaling. Up-regulated proteins in the PQ-treated rat lungs included histone cluster (HIST1H1A), adenylate cyclase (ADCY9), kinase anchor protein 6, 11 and 12 (AKAP6, AKAP11 and AKAP12, respectively), flamin A (FLNA), paxllin (PXN), inositol 1,4,5-triphosphate receptor (ITPR1), ITPR3, mitogen-activated protein kinase kinase kinase1 (MAP3K1), nitric oxide synthase (NOS3), phosphodiesterase 10A (PDE10A), PDE 3A, PDE 3B, PDE4B, and PDE4D. Down-regulated proteins in the PQ-treated rat lungs included anaphase promoting complex subunit 1 (ANAPC1), flaming B (FLNB), v-raf-1 murine leukemia viral oncogene homolog 1 (RAF1), transcription factor 4 (TCF4), transcription factor 7 like 2 (TCFTL2), protein kinase C. zeta (PRKCZ), protein kinase C. alpha (PRKCA), phosphodiesterase 12 (PDE12), inositol 1,4,5-triphosphate receptor 2 (ITPR2), β-catenin-T-cell factor and lymphoid enhancer factor complexes (TCF/LEF). In particular, the TCF/LEF complexes that are related to Wnt signaling pathway were down-regulated, while p53 binding protein 1 (TP53BP1) was not detected in the PQ-treated rat lungs.

These findings indicated that up-regulation of the canonical Wnt signaling pathway may induce cancer formation since p53-mediated Wnt repression has not taken place in the lungs and then loss of p53 function may contribute to cancer formation [41].

### P53 signaling pathway in rat lungs relevant to PQ exposure

**Figure 4:**
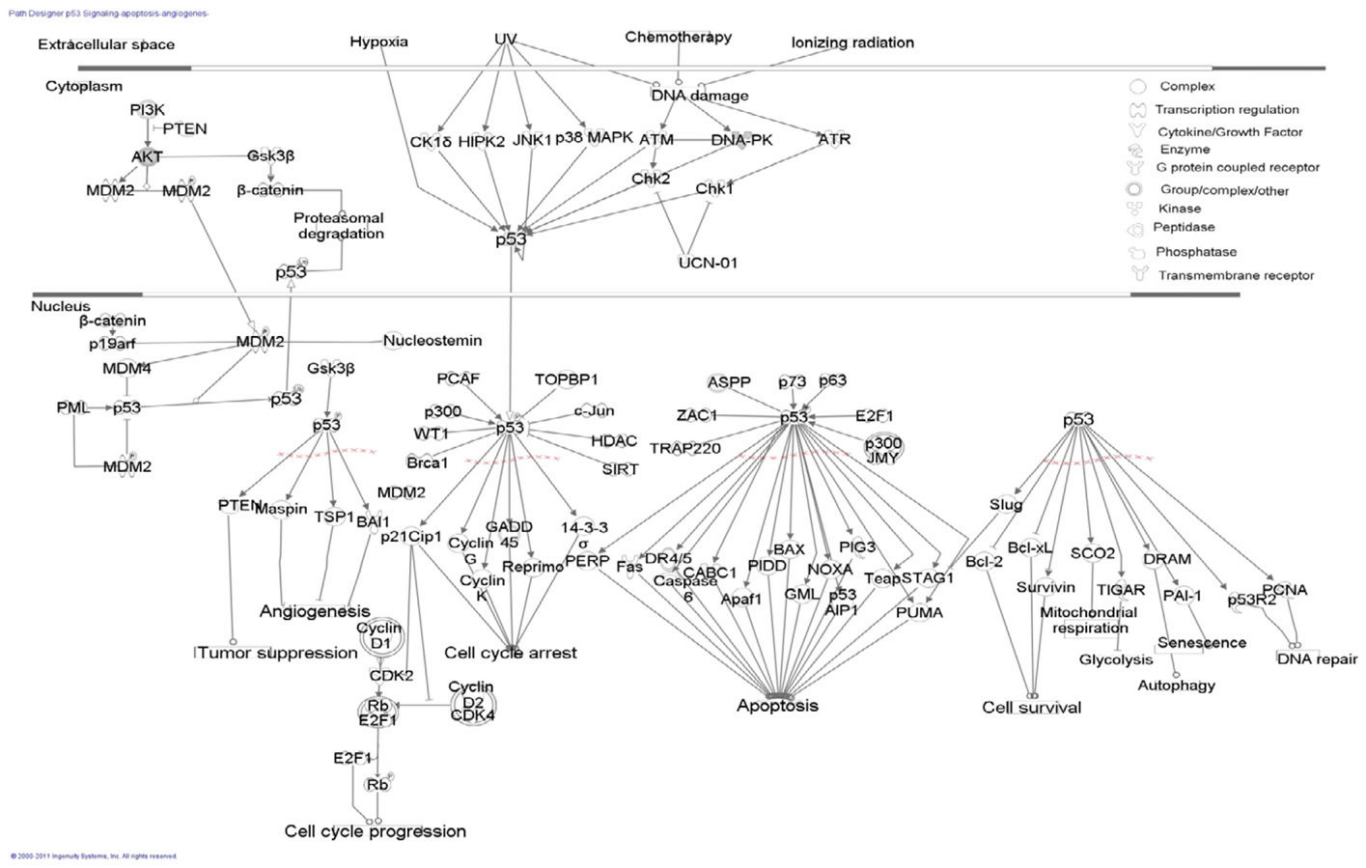
Proposed p53 signaling pathway in PQ-treated rat lungs.

The p53 tumor suppressor protects against cancer by eliminating cells that have suffered DNA damage or proliferate in an uncontrolled manner [44]. Loss of p53 function is thought to be a contributing factor in the majority of cancer cases [43]. The p53 tumor suppressor protein regulates the expression of a wide variety of genes involved in apoptosis, growth arrest, inhibition of cell cycle progression, differentiation and accelerated DNA repair or senescence in response to genotoxic or cellular stress (Figure 4) [45]. In the p53 signaling pathway, phosphate and tensin (PTEN), p53, Bcl-2, PARP apoptosis inhibitors, ABT-263, ABT-737, AZD 2281 and YM155 are apoptosis inhibitors [44,46]. Bcl-2 regulates apoptosis, which is a cell suicide program that normally responds to stresses such as growth factor deprivation, cytokine exposure, DNA damage, cell cycle dysfunction and oncogene activation [47–49]. We have found that tumor suppressor p53-binding protein 1, B-cell lymphoma/leukemia 11B, diphthamide biosynthesis protein 1, hypermethylated in cancer 1 protein, and chromosome transmission fidelity protein 8 homolog isoform 2 were down-represented in the PQ treated groups compared with the control group. These proteins are related to p53 signaling pathway that suppresses development of tumor in lungs. The results indicated that p53 signaling pathway was down-regulated through activation of Wnt signaling pathway where proteins were over-represented, which included adenomatous polyposis coli protein 2 (APC2), tuber in/tuberous sclerosis 2 (TSC2), leucine-rich repeat flightless-interacting protein 2 (LRRFIP2), dapper homolog 1 (DACT1), GSK-3-binding protein (FRAT2) and microtubule-actin cross-linking factor 1 (MACF1) in the PQ-treated rat lungs. The proteins involved in the p53 signaling pathway, MAPK signaling pathway, cartilage development and angiogenesis inhibition were under-represented in the lungs of the PQ-treated rats.

The findings indicated that PQ affected the p53 signaling pathway (Figure 4) and, thus, the impaired p53 pathway might lead to the activation of angiogenesis in lungs. PQ might cause inhibition of p53 signaling pathway, which tumor suppressor p53 binding protein 1 and B-cell lymphoma/leukemia 11B were not detected in the PQ-treated rat lungs, but detected in the control rat lungs (Table 1).

## Discussion

PQ NOEL for Sprague Dawley male and female rats was 7 mg/kg bw/day [26]. The median lethal dose of PQ for male rats was approximately 344 mg/kg bw [26,50]. In the present study, the exposure levels (5–40 mg/kg bw) resulted in no significant difference of bw gain, hematological parameters and clinic chemistry between the PQ-treated groups and the control. We hypothesized that low levels of PQ that do not cause phenotypic changes would cause differences in protein profiles and networks.

Protein profile changes in the PQ-treated rat lungs were evaluated through IPA in comparison with those in the PQ-free rat lungs. Caveolae-mediated endocytosis signaling that plays a role in various cellular processes such as endocytosis, cellular signaling and lipid recycling was up-regulated in the PQ-exposed rat lungs (Supplemental data Figure 2). Coatomer subunit gamma-2 (COPG2), EGF, EGFR, filamin A alpha (FLNA), complement decay-accelerating factor transmembrane isoform (Cd55/Daf2), integrin α 1 (ITGA1), ITGA2B, integrin α-X (ITGAX) and intersection-1/SH3 domain protein (ITSN1) are associated with caveolae-mediated endocytosis signaling. Internalization of caveolae can be induced by numerous stimuli, including insulin, EGF, hyperosmotic and oxidative stress, cholera toxin, bacteria and viruses. Upon cell detachment from the extracellular matrix (ECM), phosphorylated caveolon-1 translocates from focal adhesions to caveolae, where it induces its internalization. Putative molecular mechanisms of cancer were down-regulation of 11 proteins in the PQ-treated rat lungs (Supplemental data Figure 3). The associated proteins include RAC-β serine/threonine protein kinase (AKT), Rho guanine nucleotide exchange factor (ARHGEF), Janus kinase (JAK), low density lipoprotein receptor-related protein 6 (LRP6), protein kinase C α (PRKCA), protein kinase zeta (PRKCZ), V-raf-1 murine leukemia viral oncogene homolog 1 (RAF1), synaptic Ras GTPase activating protein 1 (SYNGAP1), mitogen-activated protein kinase kinase kinase 7-interacting protein (TAB2), transcription factor 4 (TCF4) and protein kinase DNA-activate, and catalytic polypeptide (PRKDC). Major signaling pathways involved in inter- and intracellular communication leading to malignant phenotypes include GPCR signaling, Ras/integrin signaling, Akt signaling, TGF-β/BMP signaling, Wnt signaling, Notch and Hedgehog (Hh) signaling, and death receptor signaling.

**Figure 5:**
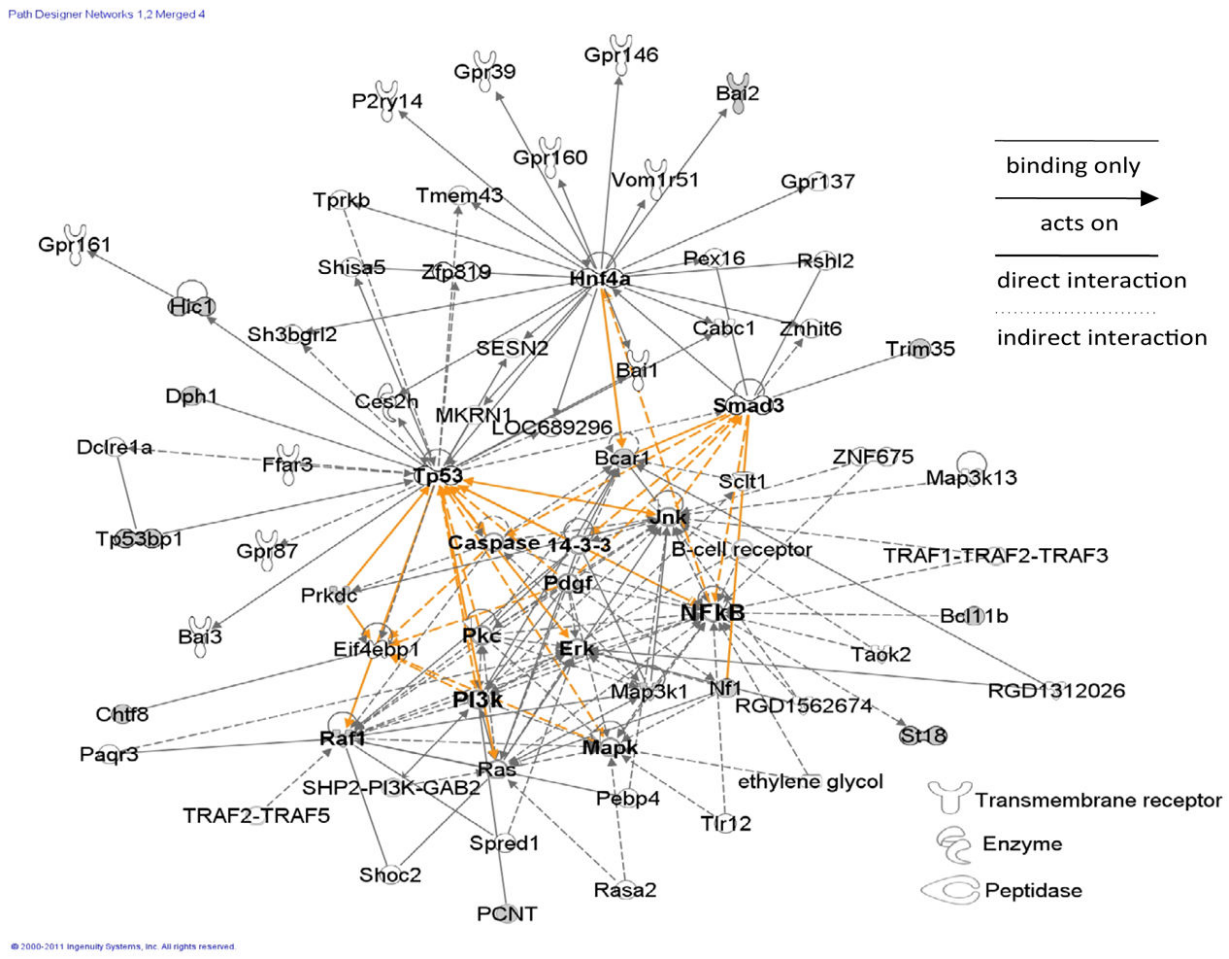
Canonical pathway and gene network analysis of the 18 identifiers that were expressed in the lungs of PQ-free rats. The 18 identifiers were analyzed using IPA and the identifiers were grouped to 11 canonical biological process pathways. The EGF signaling, PDGF signaling, NF-kB activation by viruses, ceramide signaling, regulation of IL-2 expression in activated and anergic T lymphocytes, and PTEN signaling were found to be the dominant canonical pathways represented by the identifiers. p-Value <0.05 indicated that a maximum False Discovery Rate of 5% was accepted in the functional analysis of IPA. A representative protein network of the dominant canonical pathways was then generated. The networks was obtained by merging the 2 highest scored networks into one overall network; cell signaling, cardiovascular system development (score 19), cell death (score 13). CSRNP3 and KCNH8 were not eligible to mapping. POU3F3 and EMCN were not involved in the highest scored networks. Solid grey lines and dotted grey lines indicated direct and indirect interactions, respectively. Arrow-headed lines and simple lines without arrows indicated ‘acts on’ and ‘binding only’, respectively. Solid orange lines and dotted orange lines indicated highlighted direct and indirect interactions, respectively.

The EGF signaling is the strongest functional association identified for this set of 18 identifiers (p=1×10^−3^, Fischer’s exact test). The 18 proteins that were detected in the PQ-free rat lungs, but not in the PQ-treated rat lungs (Table 1) were grouped to 11 canonical biological process pathways. Platelet-derived growth factor (PDGF) signaling, NF-kB activation by viruses, ceramide signaling, regulation of IL-2 (Interleukin 2) expression in activated and anergic T lymphocytes, phosphatase and tensin homolog (PTEN) signaling as well as the EGF signaling were found to be the dominant canonical pathways. One overall network was merged from the 2 highest scored (19, 13) networks (cell signaling; cell death) (Figure 5). Cardiovascular system development and function was the highest network identified for 18 identifiers. In the overall network, cysteine/serine-rich nuclear protein 3 (CSRNP3) and potassium voltage-gated channel subfamily H member 8 (KCNH8) among 18 identifies have not been eligible. Endomucin (EMCN) and POU domain class 3 (POU3F3) among 18 identifies have not been found to be involved in the scored (19 and 13) networks of cell signaling and cell death since the EMCN and POU3F3 belong to the cell-mediated immune response network (score, 3) and cellular growth (score, 2), respectively. Network score was based on the hyper geometric distribution and was calculated with the right-tailed Fisher’s Exact Test. The score was not an indication of the quality or biological relevance of the network; it simply measured the approximate “fit” between each network and network eligible molecules. The p-value is associated with a function or a pathway in GFA and GCP. The smaller is the p-value, the less likely random and the more significant is the association. In general, p-values less than 0.05 indicate a statistically significant, non-random association. The p-value was calculated using the right-tailed Fisher Exact Test. The pathway was activated with MAP3K1 and RAF1. These two proteins are involved in the EGF, PDGF, T cell receptor, Rac renin-angiotensin, SAPK/JNK, NF-kappa-B, ceramide signaling, and regulation of IL-2 expression in activated and anergic T lymphocytes (Figure 5).

Damage of p53-mediated apoptosis discloses a wide network of signaling pathway that is activated by p53 to make sure an appropriate response to a cellular stress [51]. p53 is known as a tumor suppressor protein encoded by the TP53 gene in humans. Some of the under-represented proteins in the network (Figure 5) were involved in the p53-signaling pathway (TP53BP1, BCL11B, DPH1 and HIC1), apoptosis (CSRNP3, PCNT and TRIM35), negative regulation of apoptosis (POU3F3 and KCNH8) and angiogenesis inhibition (BAI2 and EMCN). The suppression of tumorigenicity protein 18 (ST18) is related to the repression of basal tram inscription activity from target promoters [52]. The breast cancer anti-estrogen resistance protein 1 (BCAR1) is involved in B cell receptor signaling pathway [53].

**Figure 6:**
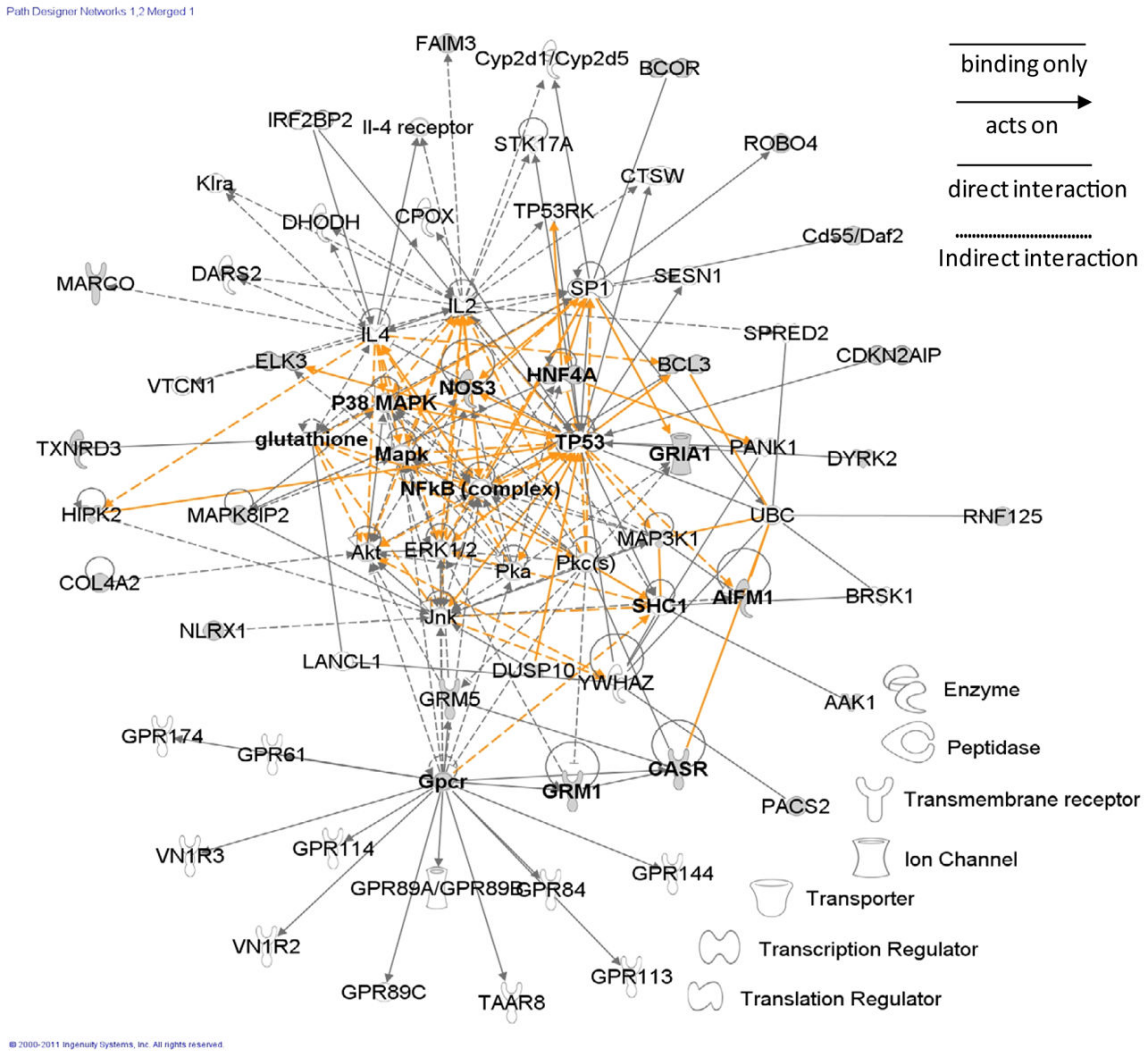
Canonical pathway and gene network analysis of the 26 identifiers that were expressed in the lungs of PQ-treated rats. The 26 identifiers were analyzed using IPA and the identifiers were grouped to 14 canonical biological process pathways. The synaptic long term depression, glutamate receptor signaling, SAPK/JNK signaling, neuropathic pain signaling in dorsal horn neurons and synaptic long term potentiation were found to be the dominant canonical pathways represented by the identifiers. A representative protein network of the dominant canonical pathways was then generated. The network was obtained by merging the 2 highest scored networks into one overall network; cell-to-signaling and interaction, nervous system development and function, cell cycle. C1orf38 was not involved in the highest scored network. Solid grey lines and dotted grey lines indicated direct and indirect interactions, respectively. Arrow-headed lines and simple lines without arrows indicated ‘acts on’ and ‘binding only’, respectively. Solid orange lines and dotted orange lines indicated highlighted direct and indirect interactions, respectively.

Synaptic long term depression was the strongest functional association identified for this set of 28 identifiers (p=1×10^−5^, Fischer’s exact test). The 26 identifiers that were detected in the PQ-treated rat lungs, but not in the PQ-free rat lungs (Table 2) were grouped to 14 canonical biological pathways. The dominant canonical pathways were the synaptic long term depression, glutamate receptor signaling, SAPK/c-Jun N-terminal kinase (JNK) signaling, neuropathic pain signaling in dorsal horn neurons, synaptic long term potentiating. One overall network was merged from the 2 highest scored (38, 25) networks (cell-to-cell signaling and interaction; cell cycle, cell-to-cell signaling and interaction) (Figure 6). Cell cycle was the highest network that was created with 26 identifiers. Among the 26 identifiers, protein THEMIS2 (C1orf38) has not been involved in the scored (38 and 25) networks since the Clorf38 belongs to the cellular development (score, 2). The pathway was activated with glutamate receptors (GRIA1, GRM1 and GRM5) and nitric oxide synthase 3 (NOS3) (Figure 6), which these proteins are involved in glutamate receptor signaling, neuropathic pain signaling in dorsal horn neurons and CREB signaling in neurons [52]. Huang et al. [55] reported that the activation of rat p38 MAPK protein increased the long term depression of synapse, which is dependent on rat GRM5 protein that was over-presented (Figure 6). Some of the expressed proteins in the network (Figure 6) were involved in the JNK pathway (DUSP10, MAPK8IP2, MAP3K1 and CASR), innate immune response (MARCO, NLRX1, FAIM3, Cd55/Daf2, BCL3 and RNF125), the macrophage inflammatory response (C1orf38) and angiogenesis (ROBO4, ELK3, COL4A2 and NOS3). The finding indicates that PQ induces oxidative stress, activation of antioxidant defense system and immune responses, and then leads to apoptotic death of neuronal cells in PQ-exposed lungs. The B-cell lymphoma 3-encoded protein homolog (BCL3) is related to T-helper 1 type immune response in nucleus as a transcriptional activator that promotes transcription of nuclear factor kappa-light-chain-enhancer of activated B cells (NF-kB) target genes. The NF-kB, controlling the transcription of DNA, is involved in cellular responses to stimuli and plays a key role in regulating the immune response to infection [56]. AP2-associated protein kinase 1 (AAK1) and NOS3 were related to the clathrin-mediated endocytosis and cardiovascular-cancer-respiratory pathway, respectively. In particular, the NOS3 pathway induces pulmonary hypertension, oxidation stress of heart. Regulation of clathrin-mediated endocytosis by AAK1 presents cell death of epithelia cell lines, cell death of kidney cell lines and cell death of embryonic cell lines [57]. CDKN2A-interacting protein (CDKN2AIP) is associated with the activation of TP53/p53 that can be induced by CDKN2Adependent and CDKN2A-independent pathways.

The findings indicated up-regulation of the neurotransmitters, JNK pathway, immune response, and angiogenesis, but down-regulation of MAPK signaling pathway, p53 signaling pathway and angiogenesis inhibition in the PQ-exposed rat lung tissues. PQ may generate ROS and impair the MAPK/p53 signaling pathway and, thus, may lead to the activation of angiogenesis and fibrosis in the lungs [58]. Tung et al. [59] reported that PQ increased connective tissue growth factor (CTGF) and collagen expression by activating the angiotensin signaling pathway in human lung fibroblasts. In the present study, proteins involved in the angiotensin signaling, connective tissue disorder and collagen were over-induced in the PQ-exposed rat lungs relative to the corresponding proteins in the PQ-free control rat lungs. The angiotensin II probably formed from angiotensin I, which has no biological activity as a precursor to angiotensin II, through removal of two C-terminal residues by the angiotensin-converting enzyme (ACE) that was conserved in the two lung groups (Supplemental data Table 6). ACE is found predominantly in the capillaries of lungs. The angiotensin II, a peptide hormone, has a role to cause blood vessels to constrict and drives blood pressure up [59]. It indicated that PQ probably inhibits production of ubiquinol. These results agreed with the report of Bus et al. [10], which PQ (30 mg/g) significantly decreased lipid-soluble antioxidant concentrations in lungs. Tomita et al. [2] reported that antioxidant defense system enzymes were up-regulated against oxidative stress in the mouse lungs and the lipid-soluble antioxidant regulated NAD(P)H dehydrogenase, quinone 1 (gene) was up-regulated. However, our work presented that NADH dehydrogenase (ubiquinone) iron-sulfur protein 8, which is related to electron transport, was found to be down-regulated in the treatment sample (Supplemental data Table 4). It is noteworthy that Kim et al. [24] reported great induction of ApoE, Pphg, Hp and C3 proteins and dramatic reduction of FGG and Ac-158 in sera upon PQ exposure, which, however, were not detected in the lung tissues in the present study (Supplementary data Table 5).

## Conclusion

There were no overt signs of toxicity based on lack of change in body weight, clinical chemistry, and hematological parameters at a dose of about 7% of the acute LD_50_. However, the expression profile of soluble proteins in the lung shows significant alterations in hundreds of proteins in the PQ-exposed (25 mg/kg) rat lung tissues in comparison with the PQ-free control rat lungs. The profile indicates that PQ induces oxidative stress and then leads to apoptotic death of neuronal cells. Eventually, activation of antioxidant defense system and immune responses in lungs after PQ exposure signify potential induction of pulmonary stresses and diseases. PQ may generate ROS and impair the MAPK/p53 signaling pathway and, thus, may lead to the activation of angiogenesis and fibrosis in the lungs.

The results of the present study were interpreted with particular emphasis on the p53 signaling pathway, which suggests PQ effects on apoptosis and angiogenesis, tumor suppression, cell cycle progression, cell cycle arrest, cell survival, autophagy, mitochondrial respiration, DNA repair, senescence and glycolysis in lungs.

## Supplementary Material

Supplementary file

## Figures and Tables

**Table 1:** Major under-represented proteins in lungs of rats that were orally administered with PQ at 25 mg/kg bw for five times a week for four weeks.

Protein name (gene symbol*)	A†	B†	C†	Biological function
**p53 signaling pathway**				
Tumor suppressor p53-binding protein 1 (*TP53BP1*)	14	160 (29)	P70399	Checkpoint signal during mitosis
B-cell lymphoma/leukemia 11B (*BCL11B*)	18	194 (29)	Q99PV8	Tumor-suppressor protein involved in T-cell lymphomas, BCL-11B (Radiation-induced tumor suppressor gene 1 protein)
Diphthamide biosynthesis protein 1 (*DPH1*)	14	142 (29)	Q5NCQ5	Tumor suppressor in lung and breast cancers
Hypermethylated in cancer 1 protein (*HIC1*)	15	160 (29)	Q9R1Y5	Tumor suppressor (Development of head, face, limbs and ventral body wall)
Chromosome transmission fidelity protein 8 homolog ( *CHTF8*)	18	158 (29)	B2RYL1	Potential tumor suppressor
**MAPK signaling pathway or related proteins**				
Mitogen-activated protein kinase kinase kinase 1 (*MAP3K1*)	14	136 (30)	Q62925	Activation of JNKK activity up
Neurofibromin 1 ( *NF1*)	14	165 (30)	P97526	MAPKKK cascade
RAF proto-oncogene serine/threonine-protein kinase (*RAF1*)	13	145 (30)	P11345	MAPKKK cascade
Suppression of tumorigenicity protein 18 (*ST18*)	13	145 (30)	Q9QX27	Negative regulation of transcription from RNA polymerase II promoter
**Apoptosis**				
Cysteine/serine-rich nuclear protein 3 (*CSRNP3*)	12	141 (30)	P59055	Apoptosis
Pericentrin (*PCNT*)	12	144 (30)	P48725	Apoptosis
Tripartite motif-containing protein 35 (*TRIM35*)	12	134 (30)	Q5RKG6	Apoptosis
POU domain, class 3 (*POU3F3*)	13	150 (30)	Q63262	Negative regulation of apoptosis
Potassium voltage-gated channel subfamily H member 8 (*KCNH8*)	12	141 (30)	Q9QWS8	Negative regulation of apoptosis
**Angiogenesis inhibition**				
Brain-specific angiogenesis inhibitor 2 (*BAI2*)	14	166 (30)	Q8CGM1	Angiogenesis inhibition
Endomucin (*EMCN*)	12	137 (30)	Q6AY82	Angiogenesis
**B cell receptor**				
Breast cancer anti-estrogen resistance protein 1 (*BCAR1*)	13	139 (30)	Q63767	B cell receptor signaling pathway
DNA-dependent protein kinase catalytic subunit (*PRKDC*)	15	150 (29)	P97313	B cell lineage commitment (serine/threonine-protein kinase)

* Protein IDs uploaded into IPA were converted to the corresponding Entrez Gene symbols for display in Pathways and Networks. Ingenuity’s knowledge base contained published findings for both genes and proteins. However, for display purposes, only gene names were used (even when protein IDs were uploaded)

† A: Number of matched peptides; B: Mascot score (p = 0.05); C: Protein accession number.

**Table 2:** Major over-represented proteins in lungs of rats that were orally administered with PQ at 25 mg/kg bw for five times a week for four weeks.

Protein (gene symbol*)	A†	B†	C†	Biological function
**c-Jun N-terminal kinase (JNK) pathway**				
Dual specificity protein phosphatase 10 (*DUSP10*)	15	143 (30)	Q9ESS0	Negative regulation of JNK cascade
C-jun-amino-terminal kinase-interacting protein 2 (*MAPK8IP2*)	18	296 (30)	Q9ERE9	The JNK-interacting protein (JIP)
Extracellular calcium-sensing receptor (*CASR*)	13	145 (30)	P48442	JNK cascade
Mitogen-activated protein kinase kinase kinase 1 (*MAP3K1*)	14	136 (30)	Q62925	Activation of JNKK activity
**Immune response**				
Macrophage receptor (*MARCO*)	14	255 (33)	Q60754	Innate immune response
NLR family member X1 (*NLRX1*)	13	251 (33)	Q5FVQ8	Innate immune response
Fas apoptotic inhibitory molecule 3 (*FAIM3*)	12	146 (32)	Q5M871	Immunity
Protein THEMIS2 (*C1orf38*)	15	141 (30)	Q91YX0	Macrophage inflammatory response
Complement decay-accelerating factor transmembrane isoform (*Cd55/Daf2*)	19	141 (33)	Q61476	Complement pathway/Innate Immunity
B-cell lymphoma 3-encoded protein homolog (*BCL3*)	15	258 (30)	Q9Z2F6	T-helper 1 type immune response
Thioredoxin reductase 3 (*TXNRD3*)	17	384 (30)	Q99MD6	Glutaredoxin
E3 ubiquitin-protein ligase RNF125 (*RNF125*)	14	252 (32)	Q9D9R0	Immune response
**Apoptosis**				
Apoptosis-inducing factor, mitochondria-associated 1 (*AIFM1*)	14	143 (30)	Q9JM53	Apoptosis
Phosphofurin acidic cluster sorting protein 2 (*PACS2*)	12	251 (32)	Q3V3Q7	Apoptosis
Dual specificity tyrosine-phosphorylation-regulated kinase 2 *DYRK2*)	11	258 **(30)**	Q5U4C9	Apoptosis
Homeodomain-interacting protein kinase 2 (*HIPK2*)	12	145 (30)	Q9QZR5	Apoptosis
**Angiogenesis**				
Roundabout homolog 4 (*ROBO4*)	13	249 (33)	Q80W87	Angiogenesis
ETS domain-containing protein (*ELK3*)	14	145 (33)	P41971	Angiogenesis
Collagen alpha-2 (IV) chain (*COL4A2*)	12	140 (30)	P08122	Angiogenesis
Nitric oxide synthase 3 (*NOS3*)	12	140 (30)	P70313	Lung development/Cardiovascular-cancer-respiratory pathway
**Neurotransmitters**				
Glutamate receptor, metabotropic 5 (*GRM5*)	13	252 (30)	P31424	Neurotransmitters
Glutamate receptor, metabotropic 1 (*GRM1)*	16	143 (29)	P97772	Neurotransmitters
Glutamate receptor, ionotropic (*GRIA1*)	12	141 (33)	P19490	Neurotransmitters
**Activation of TP53/p53**				
CDKN2A-interacting protein (*CDKN2AIP*)	8	185 (32)	Q8BI72	Activation of TP53/p53
**Chromatin modification**				
BCL-6 corepressor (*BCOR*)	13	253 (32)	Q8CGN4	Negative regulation of gene-specific transcription from RNA polymerase II promoter
**Clathrin-mediated endocytosis**				
AP2-associated protein kinase 1 (*AAK1*)	17	274 (32)	P0C1X8	Clathrin-mediated endocytosis

* the same as Table 1 footnote.

† A: Number of matched peptides; B: Mascot score (p=0.05); C: Protein accession number.

**Table 3:** Network functions, top canonical pathways, tox lists and top molecules in rat lung tissues in response to paraquat.

Up-regulation	Down-regulation
A. Top five up- and down-regulated network functions (network scores*)	
1. Cell signaling, cell death, post-translational modification (35)	1. Genetic disorder, neurological disease, cell-to-cell signaling and interaction (43)
2. Cell cycle, cellular assembly and organization, cancer (35)	2. Organism injury and abnormalities, reproductive system disease, skeletal and muscular system (41)
3. Developmental disorder, neurological disease, cardiovascular system development and function (34)	3. Endocrine system disorder, genetic disorder, metabolic disease (32)
4. Cell signaling, DNA replication, recombination, and repair, nucleic acid metabolism (30)	4. Cell-to cell signaling and interaction, tissue development, cellular movement (29)
5. Connectible tissue disorder, genetic disorder, dermatological diseases and conditions (26)	5. Dermatological diseases and conditions, genetic disorder, cancer (21)
B. Top five up- and down-regulated canonical pathways (p values†)	
1. Caveolae-mediated endocytosis signaling (2.83E-07)	1. Molecular mechanisms of cancer (1.93E-04)
2. Protein kinase A signaling (1.02E-05)	2. Acute myeloid leukemia signaling (2.95E-04)
3. EGF signaling (8.92E-05)	3. Erythropoietin signaling (1.16E-03)
4. GNRH signaling (1.67E-04)	4. Mechanisms of viral exit from host cells (1.23E-03)
5. Cardiac β-adrenergic signaling (2.69E-04)	5. IL-3 signaling (1.5E-03)
C. Top five up- and down-regulated tox lists (p values†)	
1. Increase glomercular injury (1.06E-02)	1. VDR/RXR activation (1.36E-02)
2. Hepatic fibrosis (1.2E-02)	2. G2/M DNA damage checkpoint regulation (1.9E-02)
3. Renal necrosis/cell death (3.11E-02)	3. Cardiac fibrosis (2.6E-02)
4. TR/RXR activation (4.99E-02)	4. PPARα/RXRα activation (5.11E-02)
5. RXR/RXR activation (9.64E-02)	5. p53 signaling pathway (1.88E-02)
D. Top five up- and down-regulated canonical functions	
1. Cardiovascular-cancer-respiratory	1. Epithelial cell differentiation signaling
2. Clathrin-mediated endocytosis	2. Cartilage and Cerellum and Lung development
3. Immune response and Oxidative stress and Pulmonary hypertension	3. Mitogen-activated protein kinases signaling
4. Non-small cell lung cancer	4. Transforming growth factor β (TGF-β)signaling
5. Neurotransmitter and collagen synthesis	5. Tumor suppressor and Angiogenesis inhibition

* network scores given in the parentheses were based in the hyper geometric distribution and were calculated with the right-tailed Fisher’s Exact Test. The scores were not an indication of the quality or biological relevance of the network; they simply measured the approximate “fit” between each network and the network eligible molecules.

† p values in the parentheses were associated with a function or a pathway in Global Functional Analysis (GFA) and Global Canonical Pathway (GCP); a measure of the likelihood that the association between a set of focus genes in the experiment and a given process or pathway was due to a random chance.
